# Effect of Resistance Training on Older Adults with Sarcopenic Obesity: A Comprehensive Systematic Review and Meta-Analysis of Blood Biomarkers, Functionality, and Body Composition

**DOI:** 10.3390/nursrep15030089

**Published:** 2025-03-04

**Authors:** Luis Polo-Ferrero, Víctor Navarro-López, Manuel Fuentes, Jesus Lacal, María Dolores Cancelas-Felgueras, Natalia Santos-Blázquez, Roberto Méndez-Sánchez, Juan Luis Sánchez-González

**Affiliations:** 1 Department of Nursing and Physiotherapy, University of Salamanca, 37007 Salamanca, Spain; pfluis@usal.es (L.P.-F.); nsantosblazquez@usal.es (N.S.-B.); juanluissanchez@usal.es (J.L.S.-G.); 2 Institute of Biomedical Research of Salamanca (IBSAL), 37007 Salamanca, Spain; mfuentes@usal.es (M.F.); jlacal@usal.es (J.L.); 3 Department of Physical Therapy, Occupational Therapy, Rehabilitation and Physical Medicine, Faculty of Health Sciences, Universidad Rey Juan Carlos, 28922 Madrid, Spain; victor.navarro@urjc.es; 4 Translational and Clinical Research Program, Cancer Research Center (IBMCC, CSIC-University of Salamanca), Cytometry Service, NUCLEUS, Department of Medicine, University of Salamanca, 37008 Salamanca, Spain; 5 Biomedical Research Networking Centre Consortium of Oncology (CIBERONC), Instituto de Salud Carlos III, 28029 Madrid, Spain; 6 Laboratory of Functional Genetics of Rare Diseases, Department of Microbiology and Genetics, University of Salamanca, 37007 Salamanca, Spain; 7 General and Digestive System Surgery Department, Severo Ochoa University Hospital, 28911 Leganés, Spain; mdcancelasfelgueras1@gmail.com

**Keywords:** sarcopenia, obesity, sarcopenic obesity, resistance training, nursing, aging, meta-analysis

## Abstract

**Background/Objectives**: Sarcopenic obesity (SO) is a clinical condition in which there is an excess of fat mass and a loss of muscle mass, strength, and function. Its prevalence increases with age, particularly in adults over 65 years old. However, debate persists on the definition and assessment of SO. The purpose of this review is to examine the impact of resistance training on older adults with sarcopenic obesity. **Methods**: This review included studies investigating the effects of resistance training interventions in older adults with SO. A comprehensive literature search was conducted across six databases (PubMed, SCOPUS, Cochrane Library, Embase, EBSCO, and Web of Science), yielding 1882 articles. The risk of bias in the included studies was assessed using the PEDro scale and the GRADE system. **Results**: Eleven randomized clinical trials were analyzed qualitatively and nine were analyzed quantitatively. The meta-analysis demonstrated that exercise interventions revealed the positive effects of exercise mainly on physical performance ([SMD] = 0.36, [95% CI] = 0.03, 0.69, *p* = 0.003) and body composition ([SMD] = 0.35, [95% CI] = 0.12, 0.57, *p* = 0.003), with no significant differences in biomarkers ([SMD] = 0.1, [95% CI] = −0.28, 0.49, *p* = 0.52). **Conclusions**: Resistance training benefits older adults with SO, improving body composition and physical function, whereas there were no significant differences in blood biomarkers. The present review highlights the limitations of the existing evidence base. Many included studies exhibited methodological shortcomings, necessitating the cautious interpretation of findings. Future research should prioritize rigorous study designs, including larger sample sizes and extended follow-up periods, to enhance the precision and generalizability of results.

## 1. Introduction

The world’s population is aging, and a considerable increase in the number of people over 60 years (more than 1.4 billion people) of age is expected by 2030 [1]. As a natural part of aging, individuals experience a decline in skeletal muscle mass, strength, and function, a condition known as sarcopenia [2]. Sarcopenia varies among individuals due to factors such as sedentary lifestyles [3,4]. This condition can result in reduced physical performance, limitations in mobility, an increased risk of falls and fractures, and a decreased quality of life [5]. In addition, it is associated with metabolic problems and an increased risk of insulin resistance, obesity, and type 2 diabetes [6,7]. Symptoms include muscle weakness, loss of muscle mass, fatigue, and difficulties in daily activities [2]. Diagnosing this condition can be challenging due to overlapping symptoms with other health problems and the subtle presentation of age-related changes. At this point, early detection in the community nursing setting would be of great importance and help.

From a nursing perspective, sarcopenia prevention and management, within a multidisciplinary approach, requires regular resistance training (RT) that targets muscular strength and endurance, alongside a balanced diet with sufficient protein intake and other modifiable lifestyle factors [8]. Early detection and intervention are crucial to mitigate the impact of sarcopenia and to maintain muscle health during aging [9]. Sarcopenia is closely related to obesity, both characterized by a chronic state of low-grade inflammation that affects metabolic processes, altering adipose and muscle tissue [10].

Sarcopenic obesity (SO) is a multifactorial geriatric syndrome characterized by the coexistence of excess fat mass and age-related loss of muscle mass, strength, and function [2,11]. Its prevalence increases with age, affecting approximately 11% of older adults globally [12]. SO represents a significant public health concern due to its association with an increased risk of disability, cardiovascular diseases, insulin resistance, and premature mortality [13,14]. The condition creates a synergistic effect where sarcopenia and obesity amplify each other, leading to a cycle of declining muscle function, visceral fat accumulation, and metabolic disturbances, which significantly impacts the health of affected older adults [13].

Clinical data on SO are still insufficient to support a unified definition [15]. SO is influenced by several factors, such as age, a sedentary lifestyle, unhealthy eating habits, insulin resistance, inflammation, and oxidative stress [16]. Lack of physical activity and caloric excess promote adipose hypertrophy, immune cell infiltration, and elevated cytokines, contributing to muscle atrophy [17]. Adipokines affect myocyte formation, especially in older adults [18]. High LDL and low HDL levels influence muscle health and insulin resistance associated with aging and sarcopenia [19,20,21]. SO is associated with an increased risk of chronic disease, disability, and mortality in older people [22,23,24]. Reducing body fat percentage (BF%) may be beneficial [25,26], improving the health and quality of life in older adults with SO, including cardiovascular health and insulin sensitivity [22,27]. Furthermore, it has been shown that RT can modulate inflammatory and metabolic biomarkers in older and overweight adults [28,29].

Debate continues over defining and assessing SO. A sedentary lifestyle worsens sarcopenia and obesity, often compounded by comorbidities [30]. Interventions like dietary supplements, physical activity, anabolic hormones, and antioxidants are key [31,32]. RT is especially effective in preventing and managing SO [33], with regular exercise in older adults improving muscle function, mobility, and quality of life [34,35].

While meta-analyses have explored elastic band training or combined interventions [36,37], most studies mix nutrition and exercise, obscuring the specific effects of RT [33,38,39,40]. No study has exclusively examined RT’s impact on older adults with SO. This review is unique in that it analyzes all major RT modalities and pioneers quantitative blood biomarker assessments in this population, advancing the understanding of SO’s biological mechanisms and informing targeted interventions.

Although there are meta-analyses that focus on strength training with elastic bands or compare it with other interventions [36,37], most current studies combine different intervention modalities, such as nutrition and different types of exercise, making it difficult to assess the specific effect of RT [33,38,39,40]. To date, no studies have exclusively examined the impact of RT modalities on older adults with SO compared exclusively to a control group without interventions. This meta-analysis will not only provide clear evidence of such an effect, but it is also pioneering in quantitatively analyzing the influence of RT on blood biomarkers, highlighting its innovative relevance. By investigating the biological mechanisms underlying SO, this study significantly contributes to our understanding of this condition and provides valuable insights for the development of effective interventions.

## 2. Materials and Methods

### 2.1. Data Source and Search Methods

The systematic review followed the guidelines provided by the Preferred Reporting Items for Systematic Review and Meta-analysis (PRISMA) statement [41]. To identify relevant studies, computerized databases, including Medline (PubMed), SCOPUS, Cochrane Library, Embase, EBSCO and Web of Science, were searched. A combination of keywords related to the intervention, along with Boolean operators, was used for the search strategy (Table S1). This search was conducted in July 2024. Controlled vocabulary (e.g., medical subject headings) and free-text terms were utilized, which were adjusted according to the requirements of each database. In addition, the reference lists of included studies and previously published systematic reviews were examined for further relevant data. This meta-analysis was registered in the International Prospective Register of Systematic Reviews (PROSPERO), with the registration number CRD42022380499.

### 2.2. Criteria for Considering Studies and Study Selection

We searched for articles that implemented RT intervention in older adults with obesity and sarcopenia, and that assessed the effectiveness of this intervention compared to a control intervention. The control intervention could involve another type of intervention or no intervention. Variables related to body composition, physical functioning, and biomarkers, such as HDL or LDL levels, were examined. The Population, Intervention, Comparison, Outcomes, Time, and Study design (PICOTS) framework was employed to establish the eligibility criteria for article inclusion [42].

### 2.3. Population

The studied population was adults of both sexes who were over 60 years of age with SO. There were no environmental restrictions (e.g., hospitals, nursing homes, community). We included all studies in which older adults presented a state of obesity (BF% > 30% or BMI > 30 kg/m^2^) and sarcopenia (SMI ≤ 7.76 kg/m^2^, gait speed ≤ 1 m/s, or hand grip ≤ 21 kg) have been included in this analysis.

### 2.4. Intervention

Programs in which only RT was performed were included, regardless of the type of RT: exercises with elastic bands, exercises with own weight and external weight, and exercises with guided machines. If RT was combined with other treatments, such as aerobic exercise or diet, the study was excluded.

### 2.5. Comparison

Comparison groups did not receive any treatment.

### 2.6. Outcomes

Outcome measures of functionality, body composition, and biomarkers were analyzed. Functionality was assessed via the following: the timed up and go test (TUG), chair stand test (CS), hand grip test (HG), short physical performance battery (SPPB), leg press, single-leg stance (SLS) test and chest press. Body composition was assessed via the following: BF%, the skeletal muscle mass index (SMI), appendicular muscle mass (ASM), waist circumference (WC), the waist-to-hip ratio (WHR), body weight (BW), body mass index (BMI), bone mineral density (BMD), and bone mineral content (BMC). Biomarkers were assessed via the following: interleukin-6 (IL-6), LDL, HDL, triglycerides (TG), C-reactive protein (CRP), and a homeostatic model assessment of insulin resistance (HOMA-IR).

### 2.7. Time

No temporal restrictions were applied to the duration of the intervention or outcome measures.

### 2.8. Study Design

This meta-analysis included randomized controlled trials (RCTs), focusing exclusively on RT versus no intervention in older adults with SO. Studies with combined interventions (e.g., RT + nutrition) were excluded to isolate RT’s independent effects and address the gap in current research.

### 2.9. Screening and Data Extraction

Following the retrieval and deduplication of articles from the databases, two independent reviewers (J.L.S.-G. and L.P.-F.) screened titles and abstracts using the Rayyan platform to identify studies potentially meeting the inclusion criteria. These same reviewers then independently extracted data using a standardized form, including study characteristics, participant demographics, intervention details, outcome measures, and results. Disagreements in both screening and data extraction were resolved through consensus with a third reviewer (R.M.-S.). To resolve discrepancies, reviewers compared extracted data with the original article and, when necessary, contacted study authors via email for clarification on ambiguities, such as missing data (e.g., standard deviations), unclear methods, or inconsistent reporting (e.g., varying units). This approach ensured accuracy in data extraction and interpretation. Data from studies with no author response to clarification requests were excluded from the analysis.

### 2.10. Risk of Bias and the Assessment of Methodological Quality of the Studies

A comprehensive assessment of bias risk and methodological quality was conducted using the Cochrane Collaboration’s assessment tool, which evaluates domains such as selection bias, attrition bias, blinding, and sample size [43]. The tool scrutinizes randomization processes, deviations from intended interventions, missing outcome data, outcome measurement, and selection of reported results. Overall bias was categorized as “low risk” if all domains were low, “some concerns” if at least one domain raised concerns, and “high risk” if at least one domain was high risk or multiple domains had concerns potentially affecting validity.

Methodological quality was evaluated by employing the Physiotherapy Evidence Database (PEDro) quality scale [44], which comprises 11 items with a maximum score of 10. The first item is excluded from the total score calculation, but studies failing to meet it should be excluded. Scores of 9–10 indicate excellent quality, 6–8 indicate good quality, 4–5 indicate fair quality, and below 4 indicate poor methodological quality. Throughout the analysis, discrepancies were resolved by a third investigator (R.M.-S.).

### 2.11. Overall Quality of Evidence

The quality of evidence concerning RT in individuals with SO was evaluated using the GRADE (Grading of Recommendations Assessment, Development, and Evaluation) framework [45]. This assessment categorized evidence quality as high, moderate, low, or very low, considering factors such as risk of bias, indirect evidence, inconsistency of results, imprecision of results, and potential publication bias [46]. High-quality evidence implies a high level of confidence in the agreement between the true effect and the estimated effect. Moderate-quality evidence suggests a moderate level of confidence in the estimated effect, with a possibility of significant deviation from the true effect. Low-quality evidence indicates limited confidence in the estimated effect, with the potential for substantial deviation from the true effect. Very low-quality evidence reflects low confidence in the estimated effect, with a high likelihood of a significant deviation from the true effect.

### 2.12. Data Synthesis and Analysis

The meta-analysis was carried out using Review Manager statistical software (version 5.4; Cochrane, London, UK). The effects were assessed by calculating standardized mean differences (SMDs) for change scores between the baseline and the intervention. For this analysis, sample size, mean difference, and standard deviations (SDs) were collected. When a study only provided the median, along with the first and third quartiles, these values were converted into means and SDs. When the authors reported standard errors instead of SDs, these were also transformed into SDs. If the study did not present the results, the authors were contacted to request the data. If the results were not available in this format, means and SDs were estimated from graphs (Image J software v. 1.54p; National Institute of Health, Bethesda, MD, USA). If none of these approaches were feasible, the study was excluded from the quantitative analysis, and the data were presented descriptively. If a study did not report the mean difference between pre-intervention and post-intervention in each group, it was derived from the pre- and post-intervention values. In the absence of the SD of the difference, it was imputed based on other available data: (1) using alternative measures reported in the study (e.g., confidence intervals and *p*-values, following the guidelines in Chapter 6.5.2.2 of the *Cochrane Handbook*); (2) if this was not possible, using the correlation coefficient from the most similar included study (following the principles in Chapter 6.5.2.8 of the *Cochrane Handbook*); or (3) if neither option was feasible, applying a conservative correlation coefficient of 0.5 [47,48].

The meta-analysis was conducted using the inverse variance method and a random-effects model with 95% confidence intervals, as this approach yields more conservative results in cases of heterogeneity among studies, which was anticipated. *p*-values < 0.05 were considered statistically significant. An effect size (SMD) of 0.8 or higher was classified as large, an effect size between 0.5 and 0.8 was considered moderate, and an effect size ranging from 0.2 to 0.5 was categorized small.

The overall analysis of body composition, physical functionality, and biomarkers included subgroup analyses with ≥3 studies per measure.

## 3. Results

**Figure 1:**
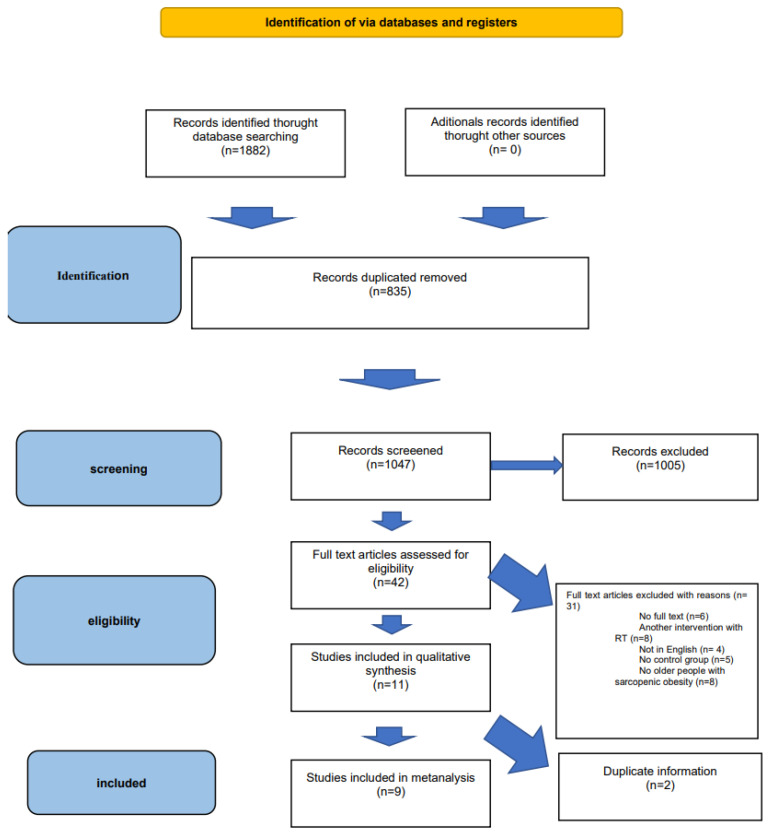
PRISMA flow diagram [41].

The initial search yielded a total of 1882 records. After removing duplicates (*n* = 1243) and conducting a title and abstract screening, 1047 records remained. Subsequently, 42 studies were considered potentially relevant, and their full reports were obtained and assessed. Out of these, 31 studies were excluded for various reasons. Finally, eleven RCTs met the inclusion criteria, of which nine were eligible for the meta-analysis, comprising a total of 513 subjects. Two articles [49,50] were excluded from the quantitative analysis, as they contained duplicate information from previous publications by the same authors [51,52]. The detailed screening process can be visualized in the PRISMA flow diagram (Figure 1).

### 3.1. Characteristics of the Included Studies

Table S2 provides a comprehensive overview of the participant characteristics in the studies included in this analysis. The selected studies primarily focused on women with SO, with one study also including both women and men. The mean age of participants across studies was 68 +/− 5.94 years. All studies involved RT, typically over a 12-week period, except for a 10-week study [53]. The frequency of the training sessions was three sessions per week, except for two sessions per week in one study [53].

### 3.2. Quality Assessment

According to the PEDro scale, the overall methodological quality of the included studies was good. One study was rated fair (score: 4–5), seven were rated good (score: 6–8), and three were rated excellent (score: 9–10) (Table S3).

### 3.3. Risk of Bias

Of the studies included, 55% demonstrated a high risk of bias, 37% had an unclear risk of bias, and 8% exhibited a low risk of bias. Among the assessed domains, the item with the highest risk of bias was related to deviations from the intended interventions, followed by missing outcome data. Conversely, the item with the lowest risk of bias was associated with the measurement of the outcome. The risk of bias values for each clinical trial can be consulted in Figure S1.

### 3.4. Quality of Evidence

The GRADE assessment indicates a moderate to very low quality of evidence for the effects of RT in older adults with SO (Table S4). Moderate-quality evidence supports improvements in body composition (SMD = 0.35; 95% CI: 0.12, 0.57). However, other outcomes, including physical performance and biomarkers, show low- to very low-quality evidence due to methodological limitations, significant inconsistencies in results, publication bias, and overall risk of bias.

### 3.5. Summary of the Included Studies

A comprehensive quantitative analysis was conducted on nine studies for each primary outcome specified in the study protocol. Subgroup analyses were performed across the categories of body composition, physical performance, and biomarker outcomes.

#### 3.5.1. Body Composition

**Figure 2:**
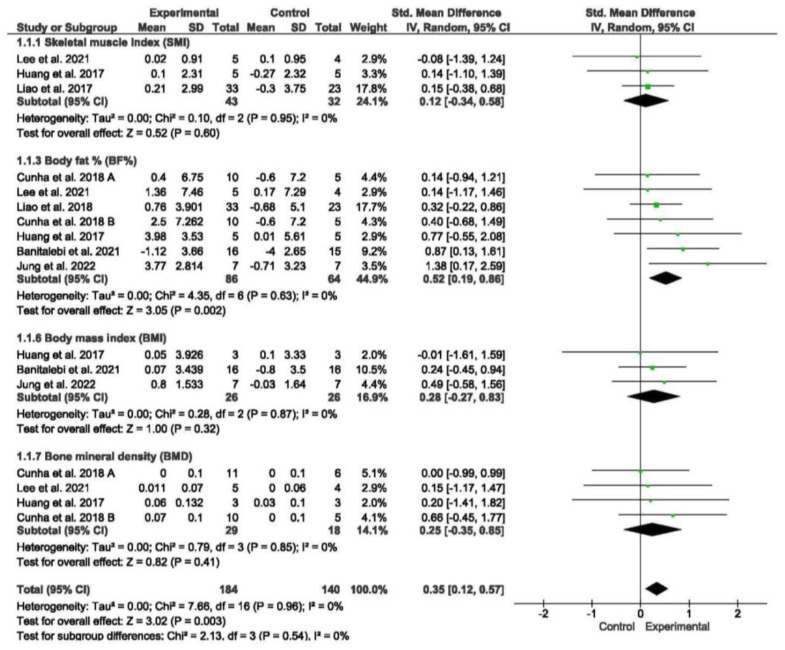
Forest plot of the results of a random-effects meta-analysis for the comparison of body composition, shown as standardized mean differences (SMDs) with 95% CIs. Shaded squares represent point estimates for each individual study and the weight of the study in the meta-analysis. Diamonds represent the overall mean difference of the studies [49,54,55,56,57,58,59].

Seven studies assessed various aspects of body composition, including the SMI, ASM, BF%, BMI, and BMD [49,54,55,56,57,58]. The meta-analysis demonstrated that exercise interventions yielded favorable effects on body composition in older individuals with sarcopenia and obesity when compared to those without exercise (standardized mean difference [SMD] = 0.35, 95% confidence interval [CI] = 0.12; 0.57, *p* = 0.003, *n* = 324) (Figure 2). Between-study heterogeneity was observed to be 0% (I^2^), indicating homogeneity between studies (*p* = 0.96). The funnel plot displayed asymmetry, suggesting a potential risk of publication bias (Figure S2).

Subgroup analyses were performed based on different body composition measures. The meta-analysis did not reveal significant differences between subgroups. However, the meta-analysis demonstrated that exercise interventions led to a significant reduction in BF% among older individuals with sarcopenia and obesity when compared to those without exercise (SMD = 0.52, 95% CI = 0.19; 0.86, *p* = 0.002, *n* = 150).

#### 3.5.2. Physical Performance

A total of four studies assessed physical performance, encompassing measures such as the timed up and go (TUG) test, gait speed, chair stand test, HG, and SLS [53,57,58,59]. Subgroup analyses were conducted for tests of gait speed, SLS, TUG, the chair stand, and HG.

**Figure 3:**
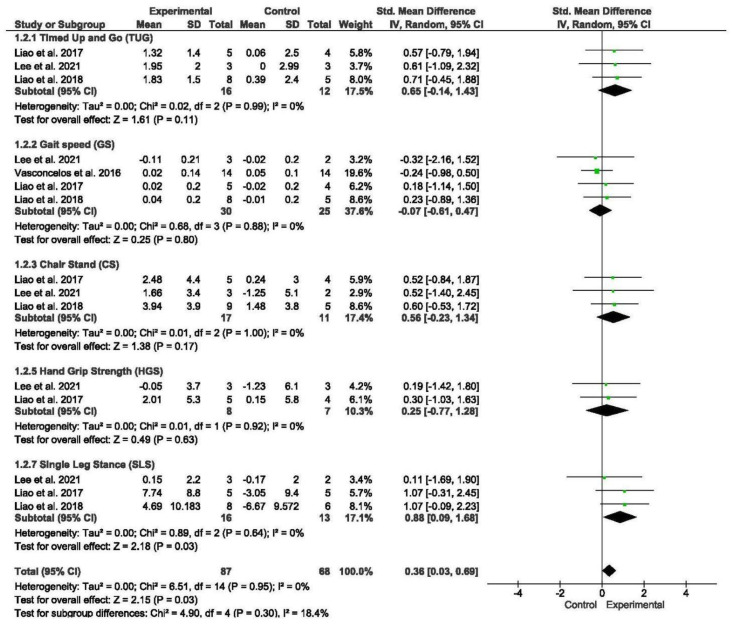
Forest plot of the results of a random-effects meta-analysis for the comparison of physical performance, shown as SMDs with 95% CIs. Shaded squares represent point estimates for each individual study and the weight of the study in the meta-analysis. Diamonds represent the overall mean difference of the studies [56,57,58,59].

The meta-analysis revealed that exercise interventions yielded positive effects on physical performance in older individuals with sarcopenia and obesity compared to those without exercise, demonstrating a low effect size (SMD = 0.36; 95% CI = 0.03; 0.69; *p* = 0.003; *n* = 155) (Figure 3). Low heterogeneity was observed among the included studies (I^2^ = 0%; *p* = 0.95). The funnel plot displayed symmetry (Figure S3).

Subgroup analyses were performed based on different measures of physical performance. The meta-analysis did not reveal significant differences between subgroups. However, the meta-analysis demonstrated that exercise interventions led to a significant decrease in SLS (SMD = 0.88; 95% CI = 0.09; 1.68; *p* = 0.03; *n* = 29) among older individuals with sarcopenia and obesity compared to those without exercise.

#### 3.5.3. Biomarkers

A total of three studies assessed biomarker concentrations, encompassing measures such as LDL cholesterol, HDL cholesterol, TG, and CRP [50,55,56]. Subgroup analyses were conducted for LDL cholesterol and HDL cholesterol.

**Figure 4:**
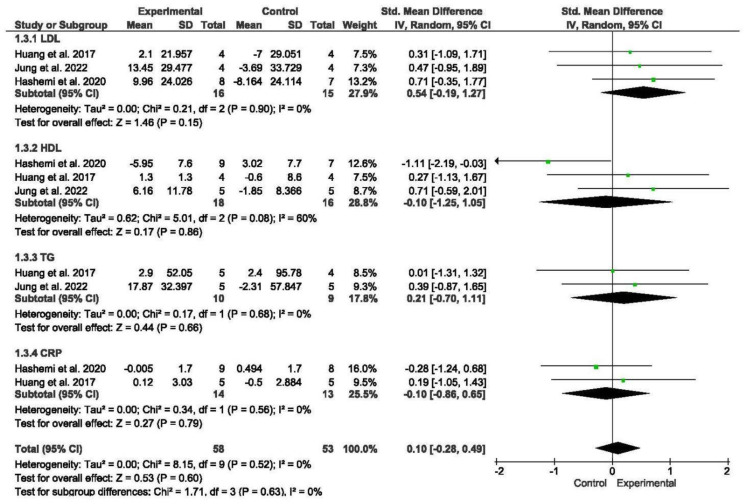
Forest plot of the results of a random-effects meta-analysis for the comparison of biomarkers, shown as SMDs with 95% CIs. Shaded squares represent point estimates for each individual study and the weight of the study in the meta-analysis. Diamonds represent the overall mean difference of the studies [52,55,56].

The meta-analysis revealed that there were no significant differences in biomarker concentrations between older individuals with SO undergoing exercise programs compared to those without exercise (SMD = 0.1; 95% CI = −0.28; 0.49; *p* = 0.6; *n* = 111) (Figure 4). Low heterogeneity was observed among the included studies (I^2^ = 0%; *p* = 0.52). The funnel plot displayed asymmetry, suggesting a potential risk of publication bias (Figure S4). Subgroup analyses were performed based on different measures of biomarker concentrations. The meta-analysis did not reveal significant differences between subgroups. 

## 4. Discussion

This meta-analysis confirms the positive effects of RT on body composition and physical performance in older adults with SO. RT significantly improved functional outcomes, particularly in the SLS test, and led to a reduction in BF%, a particularly important finding because BF% is a key diagnostic criterion for SO according to recent expert recommendations [15].

Although the observed effect sizes for body composition and physical performance were small to moderate, previous studies have demonstrated that even modest improvements in these variables can significantly reduce the risk of disability and falls in older adults [60]. The reduction in BF% is particularly relevant, given that SO is strongly associated with metabolic dysfunction and increased systemic inflammation [61]. Moreover, improvements in physical performance, particularly in balance and postural stability, are crucial for fall prevention, a major cause of morbidity in this population [62]. This underscores the need to promote resistance training programs as a part of clinical and community-based interventions for this population.

RT has been shown to be generally safe and to cause no significant adverse effects after intervention in older adults with SO, even in community-dwelling older adults participating in the intervention without direct supervision [57]. Only minor, exercise-typical adverse effects, such as muscle soreness, were reported in two of the included studies [51,53].

The findings reflect similar results to a previous study in adults with sarcopenia, evidencing the superiority of RT over other types of exercise in terms of body composition and functionality [63]. Despite the incomplete understanding of the physiological processes involved in obesity, sarcopenia, and SO, interventions targeting these processes may produce similar results [64]. Therefore, our results strongly suggest that the clinical effect of RT in adults with SO or sarcopenia may be analogous.

In individuals with obesity, studies have shown that even non-specific exercise can improve variables such as BMI, in contrast to our results [65,66].

While RT is known to enhance lean mass [29], some studies, similar to our own, have reported reductions in BF% without a corresponding increase in muscle mass [67,68,69]. Likewise, one trial found that RT did not significantly increase muscle mass in older adults with SO, but led to improvements in physical function, particularly in individuals with sarcopenia [70].

Lifestyle modification is one of the main objectives of intervention strategies in community nursing, and in generally any multidisciplinary and socio-healthcare approach, so exercise and nutrition are the two main modifiable lifestyle factors used as interventions in the treatment of SO. A low-calorie, high-protein diet has been shown to reduce BF% in older adults with SO, while supplementation has not shown the same effect. However, exercise therapy is considered a priority, as it has been shown to improve both obesity and sarcopenia, supported by scientific evidence and our own results [33]. Our study suggests that RT may be the preferred form of exercise due to its effectiveness and safety, as it has been shown to be superior to aerobic exercise in terms of functionality and body composition in adults with SO [35,71].

To better understand the effects of strength exercise in SO populations, it is essential to delve deeper into the underlying physiological processes and blood biomarkers associated with these clinical conditions. Although the involvement of biomarkers such as IL-8, TNF-α, IL-6, and IL-10 in SO-mediated processes has been established, evidence from clinical trials evaluating the impact of RT in this population is still lacking [72]. The clinical trials available to date show inconsistent results. For example, a combined aerobic exercise and RT intervention in adults with SO was found to have a beneficial effect on these biomarkers [73]. However, another clinical trial in older adults with SO, combining RT with protein supplementation, only found beneficial changes in IL-6, with no statistically significant differences in other biomarkers, such as TG, LDL, HDL, and CRP [74], which was similar to the findings of our study. On the other hand, studies have demonstrated the potential of RT to improve inflammatory and hormonal biomarker levels in older people with sarcopenia and/or obesity [75,76,77].

Regarding the analysis of the biomarkers, while this study did not yield statistically significant results, the potential positive effects of RT on key health markers cannot be entirely dismissed. It is important to consider that several factors may have influenced the lack of significant findings. For instance, the individual characteristics of the participants, such as baseline levels of inflammation or insulin resistance, may have played a role in modulating their response to RT. These factors should be carefully considered in future research to better understand how they influence biomarker outcomes.

It is crucial to recognize that RT may still positively influence these markers, as well as metabolic and hormonal health in older adults [78,79]. More research is needed to explore the potential effects of RT on relevant biomarkers and to assess its long-term impacts in older people with SO. Comprehensive research in this area may improve our understanding of the complex relationship between RT, biomarker profiles, diagnoses, and overall health outcomes in this population. This knowledge is critical for the development of specific interventions and for optimizing exercise strategies aimed at older people with SO.

This study presents some opportunities for future research and improvements in methodology. Firstly, although most studies used progressive training with elastic bands, with the exception of four studies that showed otherwise [52,53,54,57], the results suggest that strength gains with elastic bands are comparable to those obtained with conventional strength training, highlighting the versatility of elastic bands as an effective tool. Furthermore, in terms of exercise intensity, most trials progressed according to subjective feelings of exertion, using scales such as Borg, OMNI, and RPE. However, some studies also progressed according to the percentage of RM [52,53,54,55], suggesting that future research could explore more precise and controlled approaches to exercise intensity. Although this approach may introduce biases due to variability in perceived exertion among participants, it provides a sound basis for larger studies in the future. Discrepancies observed in some results could be explained by these factors, allowing for future explorations to further improve understanding of the benefits of RT in this population.

The findings on SO are mainly based on studies with a high prevalence in women, which may be related to hormonal changes affecting the development of these conditions in women [80,81,82]. However, the lack of equal representation in both genders may limit the applicability of results to a broader spectrum of SO populations. It is crucial that future research proactively addresses the inclusion of both genders in order to improve the applicability and relevance of findings for all people affected by SO.

The studies included in this analysis exhibited some heterogeneity, particularly in the methodologies used to assess physical function and biomarkers. However, body composition and physical performance outcomes showed greater consistency, with low heterogeneity reported in the meta-analysis. The variability in biomarker assessments may have influenced the overall results, making it difficult to draw definitive conclusions. Additionally, the intensity of the RT interventions may have been insufficient to induce significant metabolic changes, underscoring the need for future studies to explore higher-intensity training protocols. Similarly, the small sample sizes, the absence of blinding in outcome assessment, and the relatively short intervention duration (limited to 12 weeks in most cases) may have hindered the detection of meaningful changes in biomarkers. Longer intervention periods and standardized biomarker assessments are likely necessary to fully capture the metabolic and hormonal effects of RT.

The methodological quality and quality of evidence in many of these studies could be improved, as several of them had variations in their designs and limited methodological control, and many under-represented population diversity. To increase the reliability of findings in this field, future studies need to use more robust methodologies and standardized approaches. This article highlights the importance of improving methodological quality in SO research and encourages researchers to focus on this aspect, not only to critically evaluate findings but also to identify areas for improvement to advance the understanding of SO in older adults.

To overcome current limitations and advance the field, future research should focus on studies with larger sample sizes, longer interventions, and long-term follow-ups. Continued study of RT is essential because of its potential public health benefits, especially in the geriatric population. The identification of biomarkers relevant to the diagnosis of SO and the analysis of how exercise interventions influence these markers are key areas for future research. These advances will not only improve the understanding of this condition, but will also have crucial relevance for geriatric nursing practice. Nursing plays a central role in the promotion, implementation and supervision of adapted physical exercise programs, such as RT, which can significantly contribute to improving the quality of life of older adults [83].

This study underscores the importance of RT in geriatric nursing practice, highlighting its effectiveness in enhancing physical function and body composition in older adults with SO. The findings provide strong evidence supporting RT as a crucial intervention, directly applicable to clinical nursing practice. As sarcopenia and obesity elevate risks of disability, falls, and metabolic issues, nurses play a vital role in implementing supervised exercise programs in both clinical and community settings. By working closely with multidisciplinary teams, nurses can tailor RT interventions to patients’ specific needs, integrating them into comprehensive care plans that include health education, nutritional assessment, and personalized recommendations. This evidence-based approach not only improves the quality of life for this vulnerable population but also optimizes healthcare resources. Incorporating RT into geriatric nursing guidelines and individualizing care based on rigorous evidence promotes a proactive stance in the prevention and management of SO, ultimately enhancing overall health outcomes for older adults.

## 5. Conclusions

This meta-analysis confirms the positive effects of RT on body composition and physical performance in older adults with SO. Significant improvements were observed in BF%, a key diagnostic criterion for SO, and in physical performance measures, particularly SLS. These findings reinforce RT as an effective intervention for mitigating the functional decline associated with SO.

However, while RT demonstrates immediate benefits in physical function and body composition, its long-term effects on these outcomes, as well as on metabolic and inflammatory biomarkers, remain unclear. The lack of sustained biomarker changes suggests that longer intervention periods and follow-up assessments may be necessary to detect cumulative metabolic adaptations. Additionally, the variability in intervention protocols and biomarker assessments highlights the need for caution in interpreting these results.

To build stronger evidence, future research should focus on longitudinal studies evaluating the persistence of functional and compositional benefits over time. Studies with extended intervention durations and long-term follow-ups are required to determine whether the improvements observed in the short term translate into sustained health benefits. Furthermore, research should explore the dose–response relationship of RT and its potential synergy with other interventions, such as nutritional strategies, to optimize long-term metabolic outcomes in this population.

## Data Availability

Any data from this study required by any researcher will be made available upon request to the corresponding author. All authors had full access to all of the data (including statistical reports and tables) in the study and can take responsibility for the integrity of the data and the accuracy of the data analysis. The corresponding author had the final responsibility for the decision to submit for publication.

## Supplementary Materials

The following supporting information can be downloaded at: https://www.mdpi.com/article/10.3390/nursrep15030089/s1, Table S1: Database formulas during literature search; Table S2: Characteristics of the included studies; Table S3: PEDro Scale; Table S4: GRADE evidence for body composition, functional, and biomarker variables; Figure S1: Risk of bias; Figure S2: Body composition funnel plot. Dispersion of effect sizes; Figure S3: Physical performance funnel plot. Dispersion of effect sizes; Figure S4: Biomarkers funnel plot. Dispersion of effect sizes. File S1: PRISMA 2020 checklist.

## Guidelines and Standards Statement

This manuscript was drafted according to the PRISMA 2020 Statement.
