# Supplementary material for: Effect of Resistance Training on Older Adults with Sarcopenic Obesity: A Comprehensive Systematic Review and Meta-Analysis of Blood Biomarkers, Functionality, and Body Composition
Source: Nurs Rep. 2025 Mar 4;15(3):89. doi: 10.3390/nursrep15030089 (PMC11944422; doi:10.3390/nursrep15030089)
Supplement: Supplementary file 1 [file nursrep-15-00089-s001.zip › Figure S3. Physical performance funnel plot..pdf]

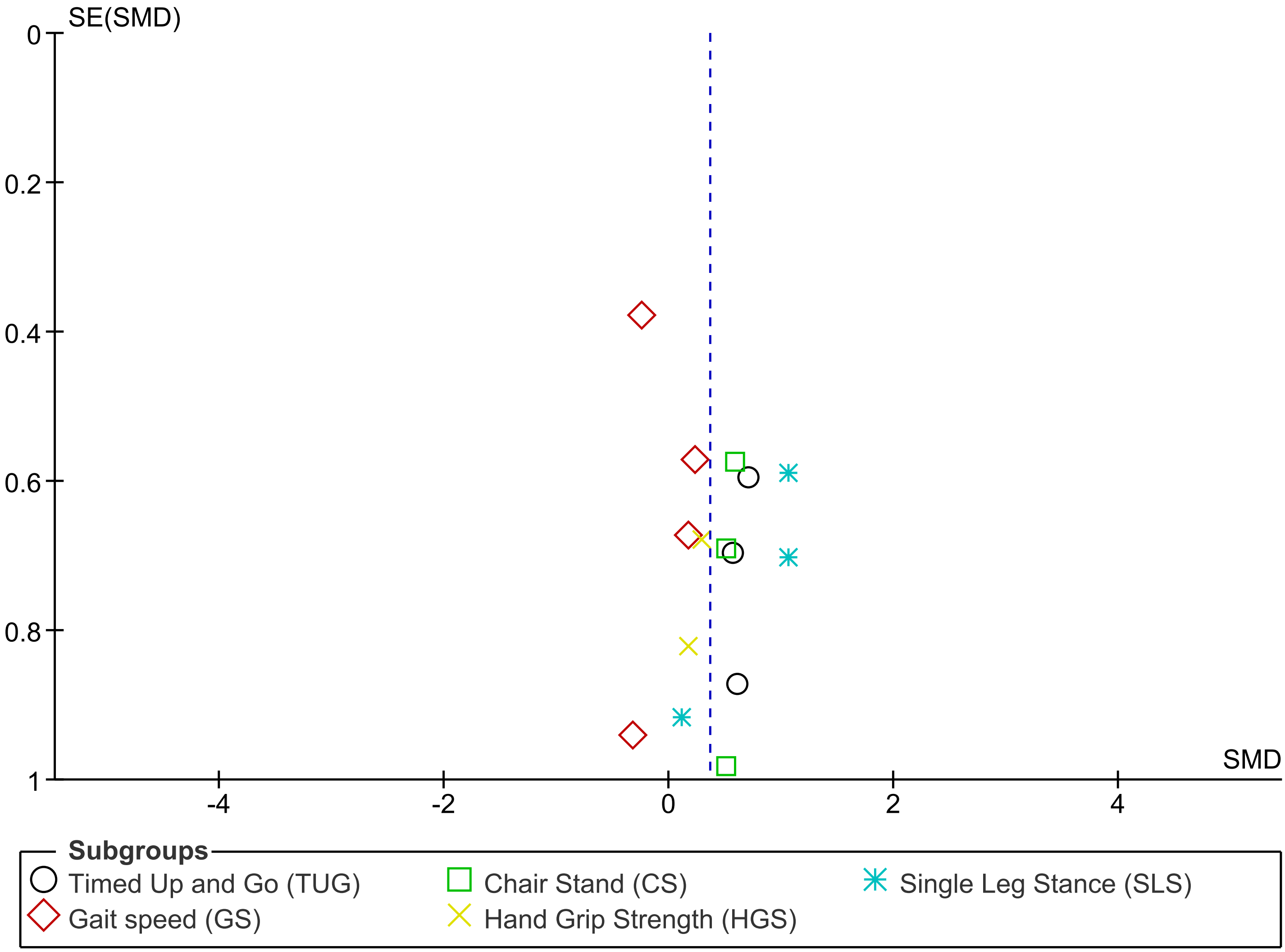
SE(SMD)
0
0.2
0.4
0.6
0.8
1
SMD
-4
-2
0
2
4
Subgroups
Timed Up and Go (TUG)
Chair Stand (CS)
Single Leg Stance (SLS)
Gait speed (GS)
Hand Grip Strength (HGS)
