# Supplementary material for: Effect of Resistance Training on Older Adults with Sarcopenic Obesity: A Comprehensive Systematic Review and Meta-Analysis of Blood Biomarkers, Functionality, and Body Composition
Source: Nurs Rep. 2025 Mar 4;15(3):89. doi: 10.3390/nursrep15030089 (PMC11944422; doi:10.3390/nursrep15030089)
Supplement: Supplementary file 1 [file nursrep-15-00089-s001.zip › Table S1. Database formulas during literature search..pdf]

nursing reports

Table S1: Database formulas during literature search

PubMed Search Formula: 322

(("RESISTANCE TRAINING" OR "STRENGTH TRAINING" OR "PROGRESSIVE RESISTANCE TRAINING" OR "STRENGTH EXERCISE" OR "RESISTANCE EXERCISE" OR "EXERCISE" OR "RESISTANCE STRENGTH TRAINING" OR "POWER TRAINING" OR "PHYSICAL EXERCISE" OR "WEIGHT TRAINING" OR "FORCE TRAINING" OR "FITNESS TRAINING" OR "PHYSICAL ACTIVITY" OR "IMPACT EXERCISE" OR "ELASTIC RESISTANCE" OR "ELASTIC EXERCISE"OR "LOADING" OR "PROGRESSIVE" OR "HIGH SPEED" OR "HIGH-SPEED") AND ("SARCOPENIC OBESITY" OR "OSTEOSARCOPENIC OBESITY" OR "OSTEOSARCOPENIC ADIPOSITY" OR "SARCOPENIC OBESE RESIDENTS" OR "SARCOPENIC OBESE ADULTS") AND (AGING OR OLD OR OLDER OR AGED)).

Cochrane Library Search Formula: 112

("RESISTANCE TRAINING" OR "STRENGTH TRAINING" OR "PROGRESSIVE RESISTANCE TRAINING" OR "STRENGTH EXERCISE" OR "RESISTANCE EXERCISE" OR "EXERCISE" OR "RESISTANCE STRENGTH TRAINING" OR "POWER TRAINING" OR "PHYSICAL EXERCISE" OR "WEIGHT TRAINING" OR "FORCE TRAINING" OR "FITNESS TRAINING" OR "PHYSICAL ACTIVITY" OR "IMPACT EXERCISE" OR "ELASTIC RESISTANCE" OR "ELASTIC EXERCISE" OR "LOADING" OR "PROGRESSIVE" OR "HIGH SPEED" OR "HIGH -SPEED") in Title Abstract Keyword AND ("SARCOPENIC OBESITY" OR "OSTEOSARCOPENIC OBESITY" OR "OSTEOSARCOPENIC ADIPOSITY" OR "SARCOPENIC OBESE RESIDENTS" OR "SARCOPENIC OBESE ADULTS") in Title Abstract Keyword AND (AGING OR OLD OR OLDER OR AGED) in Title Abstract Keyword - (Variations of the word have been searched for)

SCOPUS Search Formula: 451

(TITLE-ABS-KEY (("RESISTANCE TRAINING" OR "STRENGTH TRAINING" OR "PROGRESSIVE RESISTANCE TRAINING" OR "STRENGTH EXERCISE" OR "RESISTANCE EXERCISE" OR "EXERCISE" OR "RESISTANCE STRENGTH TRAINING" OR "POWER TRAINING" OR "PHYSICAL EXERCISE" OR "WEIGHT TRAINING" OR "FORCE TRAINING" OR "FITNESS TRAINING" OR "PHYSICAL ACTIVITY" OR "IMPACT EXERCISE" OR "ELASTIC RESISTANCE" OR "ELASTIC EXERCISE" OR "LOADING" OR "PROGRESSIVE" OR "HIGH SPEED" OR "HIGH-SPEED")) AND TITLE-ABS-KEY (("SARCOPENIC OBESITY" OR "OSTEOSARCOPENIC OBESITY" OR "OSTEOSARCOPENIC ADIPOSITY" OR "SARCOPENIC OBESE RESIDENTS" OR "SARCOPENIC OBESE ADULTS")) AND TITLE-ABS-KEY ((aging OR old OR older OR aged)))

WOS Search Formula: 749

("RESISTANCE TRAINING" OR "STRENGTH TRAINING" OR "PROGRESSIVE RESISTANCE TRAINING" OR "STRENGTH EXERCISE" OR "RESISTANCE EXERCISE" OR "EXERCISE" OR "RESISTANCE STRENGTH TRAINING" OR "POWER TRAINING" OR "PHYSICAL EXERCISE" OR "WEIGHT TRAINING" OR "FORCE TRAINING" OR "FITNESS TRAINING" OR "PHYSICAL ACTIVITY" OR "IMPACT EXERCISE" OR "ELASTIC RESISTANCE" OR "ELASTIC EXERCISE" OR "LOADING" OR "PROGRESSIVE" OR "HIGH SPEED" OR "HIGH-SPEED") (Topic) AND ("SARCOPENIC OBESITY" OR "OSTEOSARCOPENIC OBESITY" OR "OSTEOSARCOPENIC ADIPOSITY" OR "SARCOPENIC OBESE RESIDENTS" OR "SARCOPENIC OBESE ADULTS") (Topic) AND (AGING OR OLD OR OLDER OR AGED) (Topic)

Embase Search Formula: 636

(("RESISTANCE TRAINING" OR "STRENGTH TRAINING" OR "PROGRESSIVE RESISTANCE TRAINING" OR "STRENGTH EXERCISE" OR "RESISTANCE EXERCISE" OR "EXERCISE" OR "RESISTANCE STRENGTH TRAINING" OR "POWER TRAINING" OR "PHYSICAL EXERCISE" OR "WEIGHT TRAINING" OR "FORCE TRAINING" OR "FITNESS TRAINING" OR "PHYSICAL ACTIVITY" OR "IMPACT EXERCISE" OR "ELASTIC RESISTANCE" OR "ELASTIC EXERCISE"OR "LOADING" OR "PROGRESSIVE" OR "HIGH SPEED" OR "HIGH-SPEED") AND ("SARCOPENIC OBESITY" OR "OSTEOSARCOPENIC OBESITY" OR "OSTEOSARCOPENIC ADIPOSITY" OR "SARCOPENIC OBESE RESIDENTS" OR "SARCOPENIC OBESE ADULTS") AND (AGING OR OLD OR OLDER OR AGED)).

EBSCO: 497

(("RESISTANCE TRAINING" OR "STRENGTH TRAINING" OR "PROGRESSIVE RESISTANCE TRAINING" OR "STRENGTH EXERCISE" OR "RESISTANCE EXERCISE" OR "EXERCISE" OR "RESISTANCE STRENGTH TRAINING" OR "POWER TRAINING" OR "PHYSICAL EXERCISE" OR "WEIGHT TRAINING" OR "FORCE TRAINING" OR "FITNESS TRAINING" OR "PHYSICAL ACTIVITY" OR "IMPACT EXERCISE" OR "ELASTIC RESISTANCE" OR "ELASTIC EXERCISE" OR "LOADING" OR "PROGRESSIVE" OR "HIGH SPEED" OR "HIGH-SPEED")) AND (("SARCOPENIC OBESITY" OR "OSTEOSARCOPENIC OBESITY" OR "OSTEOSARCOPENIC ADIPOSITY" OR "SARCOPENIC OBESE RESIDENTS" OR "SARCOPENIC OBESE ADULTS")) AND ((AGING OR OLD OR OLDER OR AGED))

Total: 1882
