# Supplementary material for: Effect of Resistance Training on Older Adults with Sarcopenic Obesity: A Comprehensive Systematic Review and Meta-Analysis of Blood Biomarkers, Functionality, and Body Composition
Source: Nurs Rep. 2025 Mar 4;15(3):89. doi: 10.3390/nursrep15030089 (PMC11944422; doi:10.3390/nursrep15030089)
Supplement: Supplementary file 1 [file nursrep-15-00089-s001.zip › Table S2. Characteristics of the studies.pdf]

nursing reports

Table S2. Characteristics of studies

Study	Group (sample size)	Gender, Male (female)	Age, years	Sarcopenic obesity Diagnostics		Protocol intervention	Duration (weeks)	Outcome measurements
				SARC	OBE			
Banitalebi et al 2020	RT n=32	Female	64.11 ± 3.81	SMI ≤ 28% or ≤ 7,76 Kg/m2 Gait speed < 1 m/s -2.5 ≤ T-score ≤ -1	BF% >32% BMI > 30 kg/m2	RT Progressive elastic band resistance exercises 3x/wk for 12 wks. increases progressive load (OMNI scale≤ 7)	12	**Body composition** BW, BH, BMI, BF%, BMC, BMD FF, ADL **Biomarkers** EFS, Daily calorie, Carbohydrate (%), Protein (%), Vitamin D, Phosphate (mg/day)
	CG n=31	Female	64.05 ± 3.35			Received telephone contacts or face-to-face interviews on a weekly basis to maintain their typical diet and activity habits.		
Banitalebi et al 2021	RT n=32	Female	64.11 ± 3.81	(SMI) ≤ 28% or ≤ 7.76 kg/m2 gait speed (10-MWT)) ≤ 1 (m/s),	BF% > 32%, BMI > 30 kg/m2,	RT Progressive elastic band resistance exercises 3x/wk for 12 wks. increases progressive load (OMNI scale < 7)	12	**Body composition**: BH, BW, BMI, BF%, **Biomarkers** BMC, BMD, FRAX, CTX-I, miR-133, miR-206
	GC n=31	Female	64.05 ± 3.35			Telephone contacts and views on a weekly basis to maintain their typical diet and activity habits.		
Cunha et al 2018	RT 1 set n= 21	Female	66.6 ± 5.1	ASM< 17,5 Kg.	BF% > 30%	3x/wk for 12 wks. 1 set per exercise (10-15 reps)	12	**Body composition** SMM, BF%, BMD **Physical performances** Total strength
	RT 3 set n= 20	Female	68.3 ± 4.2			3x/wk for 12 wks. 3 set per exercise (10-15 reps)		
	GC n= 21	Female	67.3 ± 3.6			No exercise intervention		
Hashemi et al 2021	RT n=26	Female	64.11 ± 3.81	gait speed (10-MWT)) ≤ 1 (m/s) -2.5 ≤ T-score ≤ -1 BF% >32% by DEXA BMI >30 kg/m2		RT Progressive elastic band resistance exercises 3x/wk for 12 wks. increases progressive load (OMNI scale≤ 7)	12	**Body composition** BH. BW, BMI, BF%, BMC, BMD, **Biomarkers** hs-CRP, l HDL-C, LDL, Total Cholesterol, miR-146
	CG n=22	Female	64.05 ± 3.35			did not receive any dietary intervention or changes in their normal diet or physical		

nursing reports
MDPI

						activity during the study period.		
Hashemi et al 2022	RT n=32	Female	64.11 ± 3.81	age range of 60-80 years, BF% >32%, BMI >30 kg/m2 -2.5≤ T-score ≤-1 fr Gait speed (10 MWT) ≤ 1 m/s		RT Progressive elastic band resistance exercises 3x/wk for 12 wks. increases progressive load (OMNI scale ≤ 7)	12	**Body composition** BMI, BF% **Biomarkers** Daily calorie, Carbohydrate (%) Protein (%), Vitamin D, Calcium, Phosphate, miR-92a
	CG n=31	Female	64.05 ± 3.35			not participate in any diet or exercise programs and were instructed no to change their usual diet and physical activity during the study period.		
Huang et al 2017	RT n=18	Female	68.89 ± 4.91	SMI < 27.6%	BF% > 30%	RT Progressive elastic band resistance exercises 3x/wk for 12 wks.	12	**Body composition** BW, BH, BMI, SMI, BF%, LA fat, LA muscle, RA fat, RA muscle, Trunk fat, Trunk muscle, LL fat, LL muscle, RL fat, RL muscle, Total fat, Total muscle, Total BMD, T-score, Z-score **Biomarkers** TG, HDL, LDL, TC, GOT, GPT, BUN, Cr, CPK, CRP, Ca
	GC n=17	Female	69.53 ± 5.09			Health education booklet about SO home exercise.		
Jung et al 2022	RT n=14	Female	75.36 ± 4.50	SMI ≤ 5.4 kg/m. 2	BF% > 32%	RT (Circuit training) 3x/wk for 12 wks. Phase 1 1~2 weeks: 2 set 25 minute HRR 60~80%. Phase 2 3~8 weeks: 3 set 40 minutes Phase 3 9~12 weeks: 4 set 55 minutes	12	**Body composition** Body weight, BMI, Free fat mass (kg), Fat mass, ASM, WHR. **Biomarkers** Hs-CRP, IL-6, IGF-1, TC/HDL-C ratio, TG/HDL-C ratio, LDL-C/HDL-C ratio, ba-PWV, FPG, FPI, HOMA-IR.
	GC n=14	Female	74.64 ± 5.77			Not exercise intervention, maintaining the physical activity of your lifestyle		
Lee et al 2021	RT n=15	Female	70.13 ± 4.41		BF% > 35%	RT Progressive elastic band resistance exercises		**Body composition**: BF%, TSM, ALM, LMI, SMI

				SMI ≤ 5.67 kg/m2 And (Grip strength <20 kg or GS < 0.8 m/s		3x/wk for 12 wks. increases progressive load (Borg scale <13)	12<br>Baseline: 0 week<br>Posttest: 12 weeks<br>follow up: 36 weeks	**Physical performances**<br>HG, SLS, GS, TUG, CS<br>**Bone density parameters**<br>BMD of spine, T-score of spine
	CG n=12	Female	71.82 ± 5.23			Received group lecture and a booklet with educational content regarding SO and home exercise instructions with pictorial demonstrations.		
Liao et al 2017	RT n=25	Female	66.39±4.49	SMI <7.15 kg/m^2	BF% >30%	RT Progressive elastic band resistance exercises 3x/wk for 12 wks. increases progressive load (RPE scale <13)	12<br>Baseline: 0 week<br>Posttest: 12 weeks<br>follow up: 36 weeks	**Body composition**:<br>FFM, LLM, TFM, BF%<br>**Physical performances**<br>SLS, GS, TUG, CS, HG, LE (muscle strength)<br>**Muscle quality**<br>UE, LE,
	CG n=21	Female	68.42±5.86			No exercise intervention		
Liao et al 2018	RT n=30	Female	66.67±4.54	SMI <27.6%	BF% >30%	3x/wk for 12 wks. Progressive load (RPE scale <13)	12<br>Baseline: 0 week<br>Posttest: 12 weeks<br>follow up: 36 weeks	**Body composition**:<br>BF%, TSM, ALM, LMI, AMI, SMI (%)<br>**Functional**<br>FRD, SLS, GS, TUG, CS GPCS, SF-36-PF, SF-36-PCS,<br>**Muscle quality**<br>UE, LE.<br>.
	CG n=20	Female	68.32±6.05			No exercise intervention		
Vasconcelos et al 2016	RT n=14	Female	72±4.6	handgrip strength ≤21 kg	BMI ≥30 kg/m2	RT 2x/wk for 10 wks. Progressive load.	10	**Body composition**:<br>WC<br>**Functional**<br>SPPB, KES, KEP. GS, SF-36.
	GC N=14	Female	72±3.6			1 telephone contact/wk.		

Notes: ADL: activities of daily living; AFFM: appendicular fat free mass; ALM: appendicular lean mass; ALST: Appendicular lean soft tissue; AMI: appendicular lean mass index; ASMI: appendicular skeletal muscle mass index; ASM: Appendicular skeletal muscle mass; BF: body fat; BF%: body fat percentage; BH: body height; BMC: bone mass content; BMD: bone mineral density; BMI: body mass index; BUN; blood urea nitrogen; Ca: calcium; CPK: Creatine phosphokinase; Cr: creatinine; CS: Chair Stand; CTX-I: C-telopeptides of type I collagen; DXA: dual X-ray energy absorptiometry; EFS: Edmonton

Frail Scale; FFM: fat-free mass; FM: fat mass; FRAX: Fracture risk assessment tool; FRD: functional reach distance; FPG: fasting plasma glucose; FPI: fasting plasma insulin; GPCS: global physical capacity score; GS: gait speed; GPT: Glutamic-Pyruvic Transaminase; HDL-c: high density lipoprotein; HRR: Heart rate reserve; HP: hip circumference; Hs-CRP: High sensitivity C-reactive protein; IGF-1: insulin-like growth factor-1; KEP: knee extensor power; KES: knee extensor strength; LA: left arm; LE: lower extremity; LDL-c: low density lipoprotein; LLM: leg lean mass; LMI: lean mass index; LL: left leg; LST: lean soft tissue; miR: MicroRNA; NSAR: no sarcopenia; NC: neck circumference; OBE: obesity; PCS: physical component summary; RA: right arm; RL: right leg; RCT: Randomized Clinical Trial; RT: Resistance Training; SAR: sarcopenia; SF-36: 6-item Short Form Health Survey; SLS: Single leg stance; SM(%): Skeletal muscle %; SMM: Skeletal muscle mass; SO: Sarcopenic obesity; SPPB: short physical performance battery; TFM: total fat mass; TC: total cholesterol; TSM: total skeletal muscle mass; TUG: timed up and go test; TG: triglycerides; UE: upper extremity; WC: waist circumference; WHR: waist hip ratio; 10-MWT: 10-Meter Walk Test
