# Supplementary material for: Effect of Resistance Training on Older Adults with Sarcopenic Obesity: A Comprehensive Systematic Review and Meta-Analysis of Blood Biomarkers, Functionality, and Body Composition
Source: Nurs Rep. 2025 Mar 4;15(3):89. doi: 10.3390/nursrep15030089 (PMC11944422; doi:10.3390/nursrep15030089)
Supplement: Supplementary file 1 [file nursrep-15-00089-s001.zip › Table S3. PeDro Scale.pdf]

nursing reports

Table S3. Methodological score of clinical trials using the Physiotherapy Evidence Database (PEDro) scale.

Study	1	2	3	4	5	6	7	8	9	10	Total
Jung et al 2022	Y	Y	Y	Y	N	N	Y	Y	Y	Y	7
Huang et al 2017	Y	N	Y	N	N	Y	Y	N	Y	Y	6
Banitalebi et al 2021	Y	Y	Y	N	N	Y	Y	Y	Y	Y	8
Vasconcelos et al 2016	Y	Y	Y	N	Y	Y	Y	Y	Y	Y	9
Liao et al 2018	Y	Y	Y	N	Y	Y	Y	Y	Y	Y	9
Liao et al 2017	Y	Y	Y	Y	Y	Y	Y	Y	Y	Y	10
Lee et al 2021	Y	Y	Y	N	N	Y	Y	Y	Y	Y	8
Banitalebi et al 2020	Y	Y	Y	N	N	N	Y	Y	Y	Y	7
Hashemi et al 2021	Y	Y	Y	N	N	N	N	Y	Y	Y	6
Cunha et al 2018	Y	Y	Y	N	N	N	Y	Y	Y	Y	7
Hashemi et al 2022	N	N	Y	N	N	N	N	Y	Y	Y	4

Notes: Y > yes; N > no. (1): random allocation of participants; (2): concealed allocation; (3): similarity between group at baseline; (4): participant blinding; (5): therapist blinding; (6): assessor blinding; (7): dropout rate less than 15%; (8): intention-to-treat-analysis; (9): between-group statical comparisons; (10): point measures and variability data.
