# Supplementary material for: Effect of Resistance Training on Older Adults with Sarcopenic Obesity: A Comprehensive Systematic Review and Meta-Analysis of Blood Biomarkers, Functionality, and Body Composition
Source: Nurs Rep. 2025 Mar 4;15(3):89. doi: 10.3390/nursrep15030089 (PMC11944422; doi:10.3390/nursrep15030089)
Supplement: Supplementary file 1 [file nursrep-15-00089-s001.zip › Table S4. GRADE.pdf]

nursing reports

MDPI

Table S4. GRADE evidence for body composition, functional and biomarker variables to treat sarcopenic obesity with resistance training.

Number of studies	Risk of Bias	Inconsistency†	Indirectness‡	Imprecision§	Publication Bias¶	SMD (95% CI)	Quality of Evidence
**Body Composition**							
Seven trials (n=324)	Very serious	No Serious (I^2 = 0%)	No serious	No serious	No serious	SMD = 0.35 (0.12; 0.57)	Moderate
Skeletal Muscle Index							
Three trials (n=75)	Very serious	No Serious (I^2 =01%)	No serious	Serious	No serious	SMD = 0.12 (-0.34, 0.58)	Very Low
Body Mass Index							
Three trials (n=52)	Very serious	No Serious (I^2 = 0%)	No serious	Serious	No serious	SMD = 0.28 (-0.27, 0.83)	Very low
Body Fat %							
Seven trials (n=150)	Very serious	No Serious (I^2 = 0%)	No serious	No serious	No serious	SMD = 0.52 (0.19, 0.86)	Low
Bone Mineral Density							
Five trials (n=47)	Very serious	No Serious (I^2 = 0%)	No serious	Serious	No serious	SMD = 0.25 (-035, 0.85)	Very low
**Physical Performance**							

Four trials (n=155)	Very serious	No Serious ($I^2 = 0\%$)	No serious	Serious	No serious	SMD = 0.36 (0.03, 0.69)	Low
Chair Stand							
Three trials (n=28)	Very serious	No Serious ($I^2 = 0\%$)	No serious	Serious	No serious	SMD = 0.59 (-0.23, 1.34)	Very low
Gait Speed							
Four trials (n=55)	Very serious	Very serious ($I^2 = 0\%$)	No serious	Serious	No serious	SMD = -0.07 (-0.61, 0.47)	Very low
Hand Grip							
Two trials (n=15)	Very serious	No serious ($I^2 = 0\%$)	No serious	Serious	No serious	SMD = 0.25 (-0.77, 1.28)	very Low
Single Leg Stand							
Three trials (n=29)	Very serious	No serious ($I^2 = 0\%$)	No serious	Serious	No serious	SMD = 0.88 (0.09, 1.68)	Very Low
Timed Up & Go							
Three trials (n=28)	Very serious	No Serious ($I^2 = 0\%$)	No serious	Serious	No serious	SMD = 0.65 (-0.14, 1.43)	Very low
**Biomarkers**							
Three trials (n=111)	Very serious	No Serious ($I^2 = 0\%$)	No serious	Serious	No serious	SMD = 0.1 (-0.28, 0.49)	Very low

HDL							
Three trials (n=34)	Very serious	Very serious (I^2 = 60%)	No serious	Serious	No serious	SMD = -0.10 (-1.25, 1.05)	Very low
LDL							
Three trials (n=31)	Very serious	Serious (I^2 = 0%)	No serious	Serious	No serious	SMD = 0.54 (-0.19, 1.27)	Very low
TG							
Two trials (n=27)	Very serious	Serious (I^2 = 0%)	No serious	Serious	No serious	SMD = -0.1. (-0.28, 0.65)	Very low

Notes: GRADE ¼ Grading of Recommendations Assessment, Development and Evaluation; MD ¼ mean difference.
*"No" ¼ most information is from results at low risk of bias; "Serious" ¼ crucial limitation for one criterion or some limitations for multiple criteria sufficient to lower confidence in the estimate of effect; "Very serious" ¼ crucial limitation for one or more criteria sufficient to substantially lower confidence in the estimate of effect.
† "Serious" ¼ I^2 > 40%; "Very serious" ¼ I^2 > 80%.
‡ No indirectness of evidence was found in any study.
§ Based on sample size. "Serious" ¼ n<250 subjects; "Very serious" ¼ n<250 and the estimated effect is little or absent.
¶ Based on funnel plots. No publication bias was found. Funnel plots are not shown because the number of trials was less than 10.
