# Supplementary figures and images for: Effect of Resistance Training on Older Adults with Sarcopenic Obesity: A Comprehensive Systematic Review and Meta-Analysis of Blood Biomarkers, Functionality, and Body Composition
Source: Nurs Rep. 2025 Mar 4;15(3):89. doi: 10.3390/nursrep15030089 (PMC11944422; doi:10.3390/nursrep15030089)

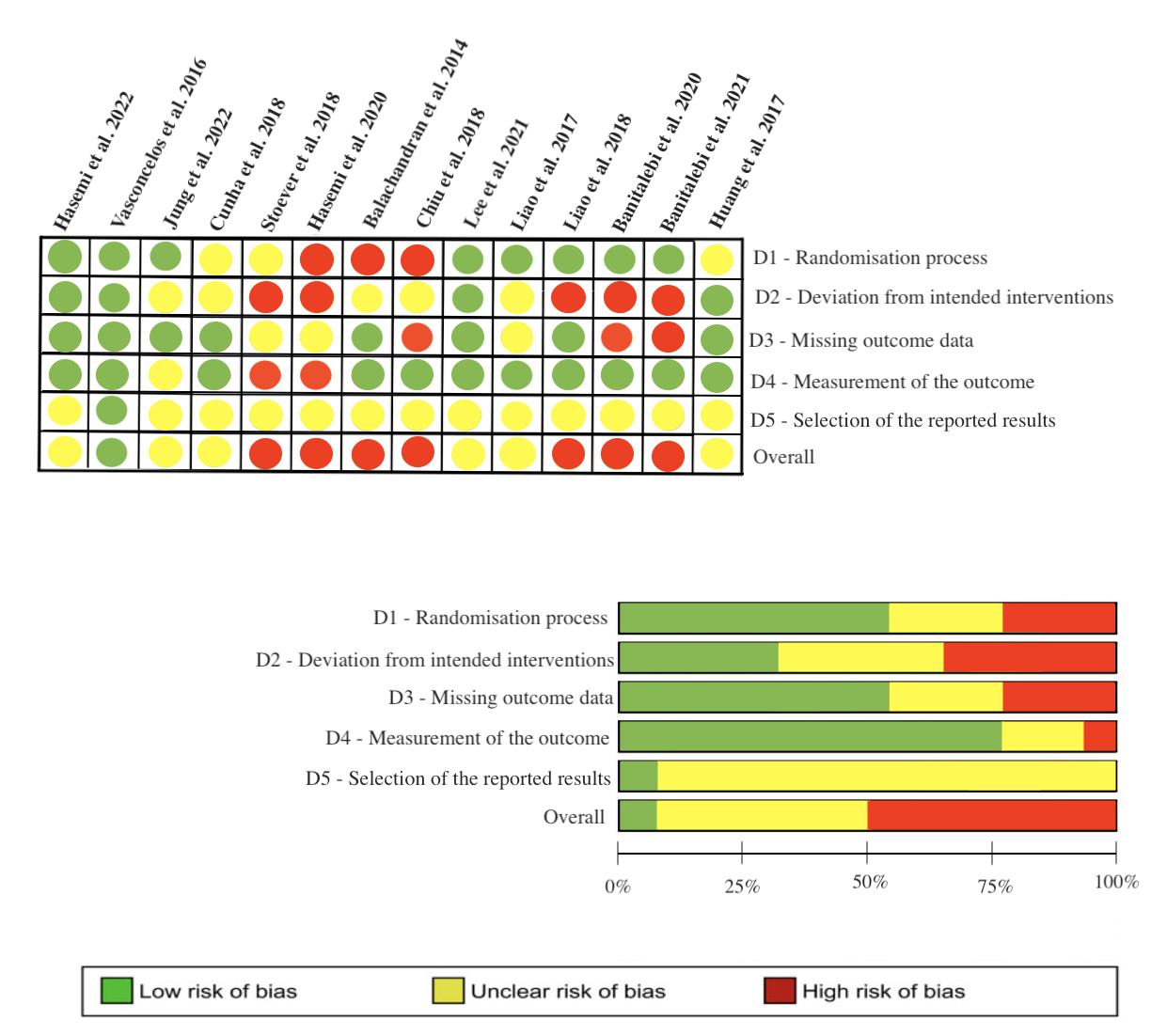

Supplement: Supplementary file 1 [file nursrep-15-00089-s001.zip › Figure S1. Risk of bias.tiff]

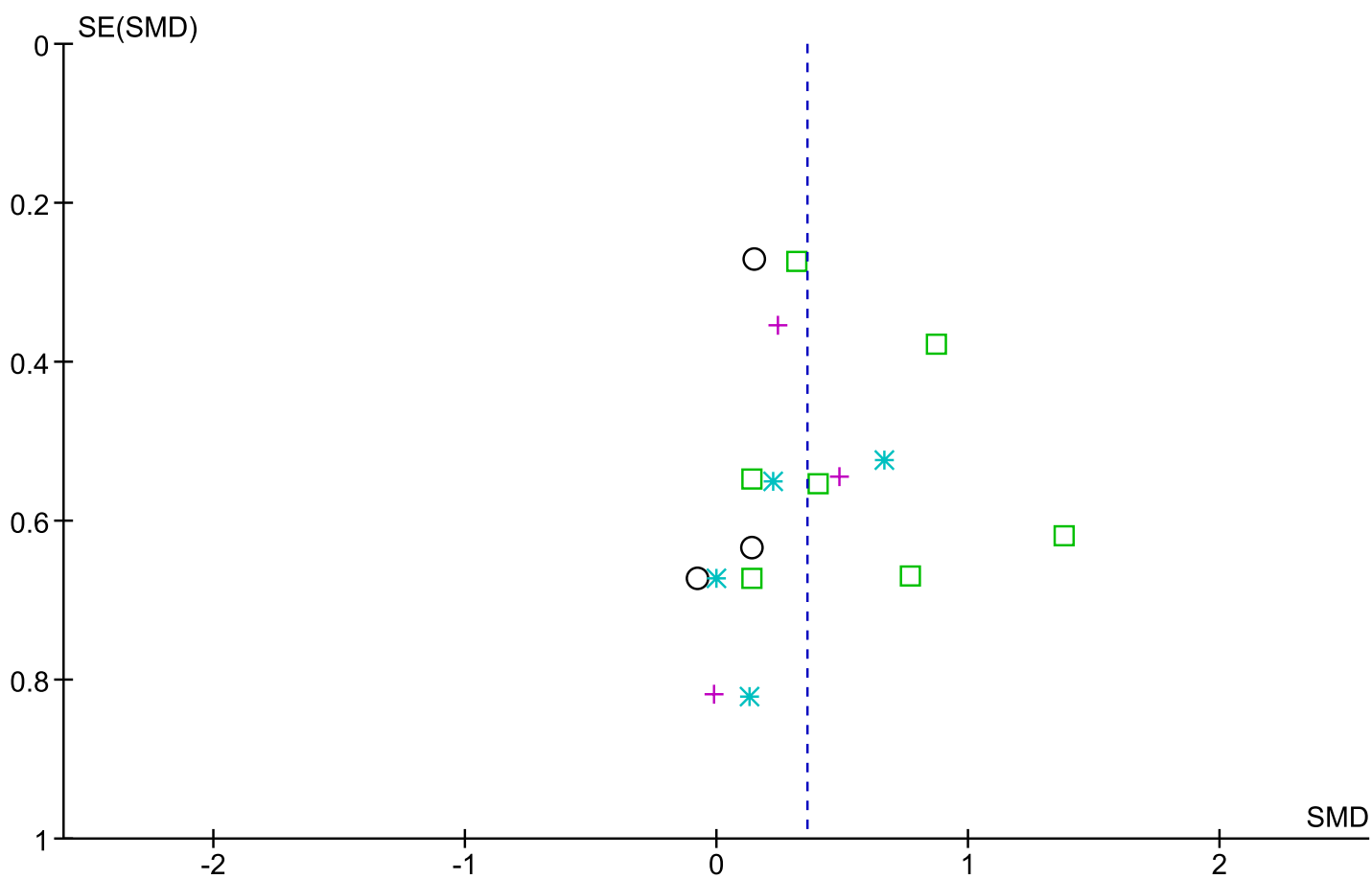

**Subgroups**

○ Skeletal muscle index (SMI)

□ Body fat % (BF%)

+ Body mass index (BMI)

\* Bone mineral density (BMD)

Supplement: Supplementary file 1 [file nursrep-15-00089-s001.zip › Figure S2. Body composition funnel plot..pdf]

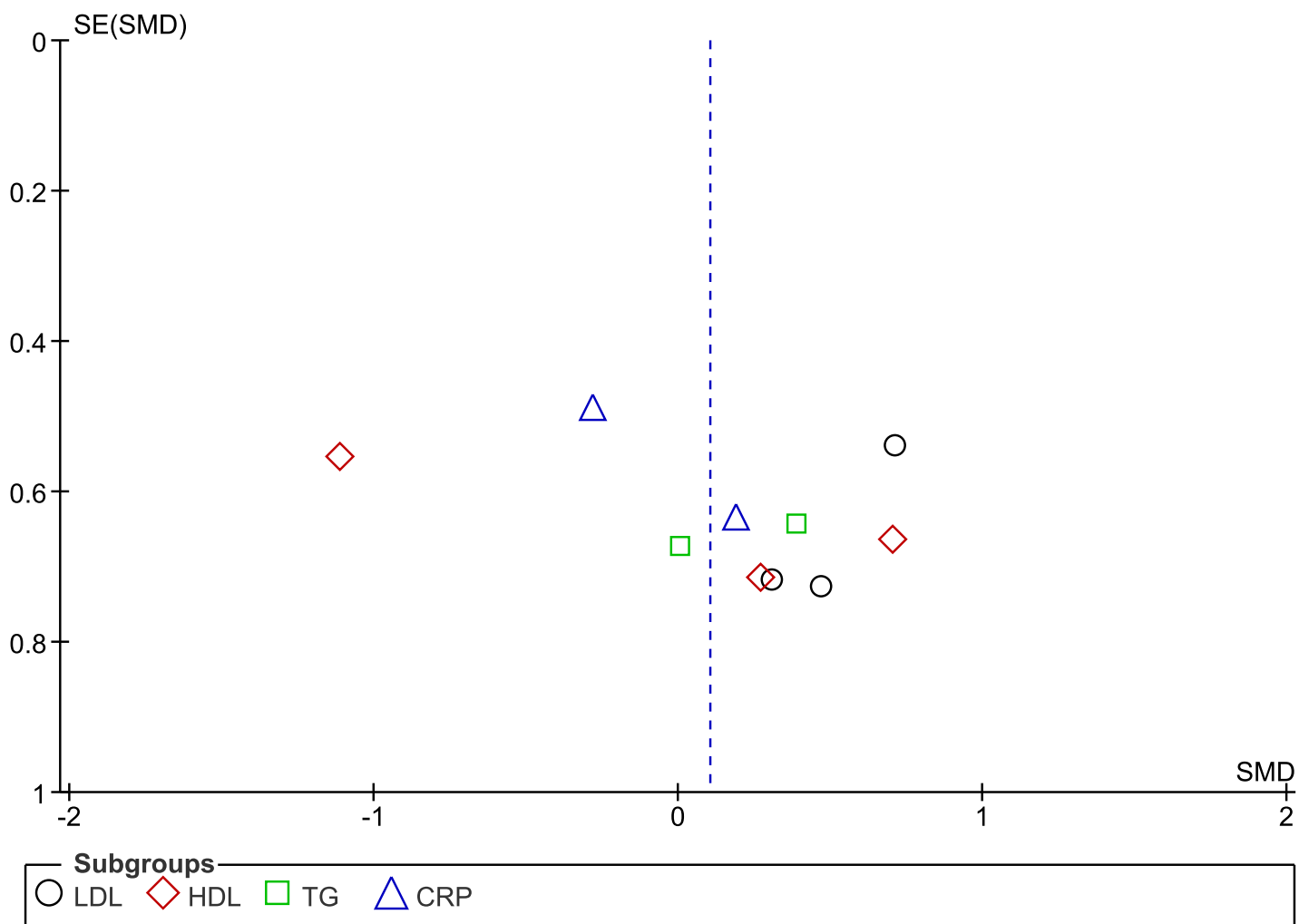

Supplement: Supplementary file 1 [file nursrep-15-00089-s001.zip › Figure S4. Biomarkers funnel plot..pdf]
